# Meprin proteases in cutaneous homeostasis and psoriasis: pathophysiological mechanisms and therapeutic prospects

**DOI:** 10.3389/fimmu.2026.1817338

**Published:** 2026-07-08

**Authors:** Vineetkumar Pillai, Mahaboobkhan Rasool

**Affiliations:** Immunopathology Lab, School of Biosciences and Technology, Vellore Institute of Technology (VIT), Vellore, Tamil Nādu, India

**Keywords:** cytokine activation, ECM remodeling, extracellular proteolysis, inflammation, meprin metalloproteinases, psoriasis

## Abstract

Psoriasis is a chronic immune-mediated inflammatory disorder characterized by epidermal hyperplasia, barrier dysfunction, and dysregulated crosstalk between keratinocytes and adaptive immune cells. Although the IL-23/IL-17 axis remains central to disease pathogenesis, the extracellular mechanisms that sustain and amplify inflammation are not well defined. Emerging evidence highlights extracellular proteolysis as a critical regulator of inflammatory persistence in psoriatic skin by modulating cytokine bioavailability, receptor activation, extracellular matrix remodeling, and immune cell trafficking, including the release of angiogenic mediators such as VEGF. Zinc-dependent metalloproteases are key contributors to these processes. Among them, meprin α and meprin β exhibit distinct structural and spatial characteristics, positioning them as context-dependent regulators of epithelial and immune homeostasis. In psoriatic lesions, meprin α overexpression results in keratinocyte hyperproliferation, barrier deficiencies with increased transepidermal water loss, and local inflammation due to the proteolytic cleavage of dermokine, an essential regulator of immune responses and epidermal differentiation. Additionally, it is mislocalized to the suprabasal layers. Meprin-α has also been shown to synchronize the activation of skin barrier proteins, effectively contributing to sustained barrier integrity. This, together with the pathogenic repercussions of the meprin-driven pro-inflammatory milieu, has far-reaching implications for this understudied protein. Thus, the current review synthesizes evidence implicating meprins in keratinocyte differentiation, barrier integrity, cytokine processing, and matrix remodeling. We further discuss the regulatory mechanisms that control meprin expression and evaluate its therapeutic potential as an upstream modulator of extracellular inflammatory signaling in psoriasis.

## 1 Introduction

Chronic plaque psoriasis (psoriasis vulgaris) is an immune-mediated inflammatory skin disease characterized by epidermal hyperproliferation, aberrant keratinocyte differentiation, angiogenesis, and sustained immune activation (1). Affecting more than 125 million individuals worldwide, psoriasis represents a complex interplay between genetic susceptibility, environmental triggers, and dysregulated immune responses (2). In addition to its known immunological basis, psoriasis is increasingly recognized as a disorder of impaired tissue homeostasis, in which alterations to the epidermal degradome—the comprehensive system of extracellular proteases, their substrates, and endogenous inhibitors—actively facilitate disease progression (3, 4). The degradome influences the structural and inflammatory characteristics of psoriatic lesions by modulating cytokine bioavailability, extracellular matrix turnover, epithelial differentiation, and immune cell trafficking (3, 5). Thus, dysregulated proteolysis is considered a significant contributor to cutaneous disease rather than merely a secondary effect of inflammation (6).

The immunopathology of psoriasis involves significant interaction between keratinocytes and infiltrating immune cells, leading to activation of Th1- and Th17-associated pathways and overproduction of inflammatory mediators such as TNF-α, IL-17, IL-23, and IL-22. These mediators drive keratinocyte activation, amplify inflammatory signaling, and remodel the skin’s extracellular microenvironment (7–10).

Psoriasis is marked by significant remodeling of the epidermal microenvironment, including immunological dysfunction. Changes in keratinocyte development, basement membrane structure, cell–cell adhesion, and barrier integrity together lead to the creation and persistence of lesions. Extracellular proteases partially govern these processes by coordinating dynamic interactions among structural proteins, cytokines, growth factors, and immune cells. Therefore, changes in proteolytic activity can simultaneously affect inflammatory reactions and epidermal homeostasis (11, 12).

Extracellular proteolysis is a vital yet often overlooked regulator of psoriatic pathology, governing cytokine maturation, extracellular matrix (ECM) remodeling, keratinocyte proliferation, immune cell migration, angiogenesis, and immune-epithelial interactions, thereby shaping both the structural and inflammatory features of lesional skin. Matrix metalloproteinases (MMPs), along with A Disintegrin and Metalloproteinase (ADAM) proteases and their endogenous inhibitors, tissue inhibitors of metalloproteinases (TIMPs), form a tightly regulated network that synchronizes cytokine signaling with ECM turnover, epithelial renewal, and vascular restructuring. Under physiological conditions, these proteases are precisely regulated; however, reviews by Park et al. (6) and Mezentsev et al. (13) indicate that psoriasis is marked by an imbalance between proteases and their inhibitors, resulting in excessive ECM degradation, basement membrane destabilization, activation of pro-inflammatory pathways, and persistent inflammation that disrupts normal tissue architecture (**Table 1****).**

**Table 1:** Role of different Matrix Metalloproteinases in normal physiological conditions vs in psoriasis.

Sr. no.	MMP	Normal physiological role	Role in psoriasis
1.	**MMP-1** (Collagenase-1)	Degrades fibrillar collagens (types I, II, III); essential for controlled dermal remodelling and wound healing	Overexpressed in psoriatic epidermis; promotes excessive collagen degradation, keratinocyte migration and plaque development
2.	**MMP-2** (Gelatinase-A)	Degrades type IV collagen and gelatin; involved in basement membrane remodelling, angiogenesis and tissue repair	Elevated in psoriatic skin; facilitates immune cell infiltration and pathological angiogenesis
3.	**MMP-3** (Stromelysin-1)	Degrades proteoglycans, laminin and fibronectin; activates other MMPs during physiological remodelling	Upregulated in psoriasis; amplifies ECM degradation and inflammatory tissue damage
4.	**MMP-7** (Matrilysin-1)	Processes ECM components and antimicrobial peptides (AMPs); supports epithelial repair and innate immunity	Increased expression enhances keratinocyte proliferation and inflammatory signalling in psoriatic lesions
5.	**MMP-8** (Collagenase-2)	Neutrophil derived enzyme involved in collagen degradation during acute inflammation and resolution	Elevated due to neutrophil infiltration in psoriatic plaques, contributing to persistent inflammation
6.	**MMP-9** (Gelatinase-B)	Regulates leukocyte migration, ECM turnover and angiogenesis during wound healing	Strongly overexpressed in psoriasis; drives immune cell infiltration, angiogenesis and epidermal hyperplasia
7.	**MMP-10** (Stromelysin-2)	Supports epithelial repair and activates other MMPs in controlled tissue remodelling	Upregulated in psoriatic epidermis, promoting keratinocyte migration and inflammation
8.	**MMP-12** (Macrophage Elastase)	Degrades elastin; regulates macrophage migration and immune responses	Increased macrophage derived MMP-12 sustains chronic inflammation in psoriatic lesions
9.	**MMP-13** (Collagenase-3)	Degrades collagen type II and III; involved in tissue remodelling and development	Elevated in psoriatic skin; contributes to severe ECM disruption and lesion persistence
10.	**MMP-14** (MT1-MMP)	Membrane type MMP is involved in pericellular matrix remodelling and activation of MMP-2	Promotes invasive keratinocyte behaviour and immune cell migration in psoriasis
11.	**MMP-15 / MMP-16** (MT2/MT3-MMP)	Regulate localized ECM remodelling and cell migration	Dysregulated expression contributes to ECM degradation in psoriatic lesions
12.	**MMP-19**	Involved in epidermal differentiation and maintenance of skin homeostasis	Altered expression disrupts keratinocyte differentiation in psoriasis
13.	**MMP-28** (Epilysin)	Expressed in normal epidermis; involved in wound healing and epithelial integrity	Dysregulation may impair barrier function and promote chronic inflammation in psoriasis

MMP, Matrix Metalloproteinases.

Meprins are among the proteases that make up the epidermal degradome, and they have recently come to light, particularly as interesting modulators of cutaneous biology. Meprins are zinc-dependent metalloproteases that belong to the astacin family and are structurally distinct from classical MMPs. The two isoforms, meprin α (*MEP1A*) and meprin β (*MEP1B*), exhibit distinct compartmentalization and substrate preferences, enabling them to regulate matrix architecture and inflammatory signaling (13–15). These characteristics put them at the nexus of inflammatory signaling, epidermal differentiation, and tissue remodeling.

Mechanistically, meprins contribute to immune activation by directly processing cytokines. Meprin β activates pro-IL-1β and pro-IL-18 independently of canonical inflammasome-mediated caspase-1 pathways, thereby establishing an alternative route for cytokine maturation (16, 17). Both meprin α and meprin β have been shown to shed the IL-6 receptor, facilitating IL-6 trans-signaling (5, 16). Additionally, meprin cleavage of ECM components, such as collagen IV, laminins, and adhesion molecules, influences basement membrane integrity and leukocyte migration (18–21).

Recent developments in proteomic and degradomic analyses have significantly broadened the range of meprin substrates identified in the skin (22, 23). Interestingly, Dermokine (DMKN) has emerged as a potential epidermal substrate whose proteolytic processing may influence inflammatory responses, keratinocyte differentiation, and barrier maintenance. These findings imply that meprins may have more extensive regulatory roles in the epidermal degradome than previously thought (24).

Emerging evidence indicates that meprins may exert significant roles within psoriatic skin. The proteolytic processing of DMKN and other keratinocyte-associated substrates involved in epidermal development and barrier maintenance has been linked to meprin α. Moreover, meprin-mediated activation of IL-1β and IL-18, IL-6R shedding, and ECM remodeling provide multiple molecular pathways through which these enzymes may combine inflammatory amplification with epithelial failure. Given their ability to integrate cytokine activation, matrix remodeling, angiogenic signaling, and epithelial differentiation, meprins are increasingly recognized as non-redundant contributors to inflammatory tissue pathology. Initial experimental investigations revealed that meprins are prevalent metalloproteases in polarized epithelial tissues, exhibiting notably high expression in the brush-border membranes of proximal renal tubules and intestinal epithelial cells (25). Unlike MMPs, whose functions often overlap, meprins exhibit distinct substrate selectivity and spatial localization, suggesting they may complement or amplify traditional metalloprotease activity within psoriatic lesions (14, 16, 19, 26).

Collectively, these observations indicate that extracellular proteolysis extends beyond classical MMP-driven matrix degradation and encompasses a broader protease network in which meprin occupies an important position. In this review, we synthesize the role of meprin proteases in the epidermal degradome and assess how dysregulated expression and activity of these proteases may influence the pathophysiology of psoriasis. We further critically evaluate emerging evidence linking their activity to key pathological features of psoriasis, including cytokine activation, leukocyte recruitment, angiogenesis, barrier dysfunction, and chronic inflammation. By integrating evidence from mechanistic studies, degradomic analyses, and experimental models, we highlight the therapeutic potential of meprins as targets for future psoriasis interventions.

## 2 Biogenesis, structural organization, and cutaneous expression of meprins

Meprins are zinc-dependent metalloproteinases of the astacin family within the metzincin superfamily (27, 28). and constitute a distinct class of proteases that operate at the epithelial and stromal interfaces (14). Unlike many extracellular metalloproteinases that function primarily as terminal degradative enzymes, meprins exhibit tightly regulated biogenesis and spatially restricted expression, particularly evident in barrier tissues such as skin (19). In mammals, these are encoded by two homologous genes, *MEP1A* and *MEP1B*, which encode the α and β subunits, respectively, and assemble into homo- or hetero-oligomeric complexes with divergent cellular localization, activation mechanisms, and substrates (23, 28–30). The skin is a key site where this molecular diversification is translated into spatially and temporally controlled proteolytic activity.

Both meprin subunits are synthesized as multidomain zymogens, the architecture of which underlies their biogenesis and function. The core catalytic domain adopts the conserved astacin fold, with a zinc ion coordinated by a canonical HEXXHXXGXXH motif and stabilized by the characteristic methionine (Meth) turn, placing meprins within the metzincin lineage (28, 31). N-terminal pro-domains maintain enzymatic latency during biosynthesis, preventing proteolysis via the secretory pathway (14). C-terminal to the catalytic domains, meprins harbor multiple regulatory modules, including MAM, TRAF-like, and EGF-like domains, which govern oligomerization, substrate recognition, and protein-protein interactions (29, 32). For instance, the MAM and TRAF-like domains of meprin β facilitate interactions with extracellular matrix substrates, such as procollagen I and collagen IV (33), promoting their proteolytic processing and contributing to matrix remodeling in skin and kidney tissues. The EGF-like domains further support substrate positioning and receptor interactions, enabling the cleavage of membrane-associated proteins, including E-cadherin and the IL-6 receptor (15, 23, 34), thereby influencing epithelial barrier integrity and inflammatory signaling. In addition, meprin-mediated processing of pro–IL-1β demonstrates how structural organization directly determines cytokine activation and downstream inflammatory responses in the cutaneous environment (35). Foundational biochemical studies established that meprin α and meprin β assemble into homo- and hetero-oligomeric complexes whose structural organization dictates enzyme localization, substrate accessibility, and biological function. These studies further demonstrated that both isoforms are synthesized as inactive zymogens requiring proteolytic removal of N-terminal propeptides for activation. Subsequent post-translational processing determines whether the enzyme remains membrane-associated or is released into the extracellular environment, thereby generating spatially restricted proteolytic activity within epithelial tissues (29). Following translation, the meprin polypeptides enter the endoplasmic reticulum, where folding, N-linked glycosylation, and disulfide bond formation occur before oligomerization. These steps are critical determinants of downstream trafficking and activities. Meprin subunits assemble into disulfide-linked dimers and higher-order complexes before transit through the Golgi apparatus, where post-translational processing diverges sharply between α and β subunits (19).

A defining feature of meprin β is the presence of a transmembrane domain and a short cytoplasmic tail, which anchor it to the plasma membrane and confine its proteolytic activity to the pericellular region. Conversely, meprin α contains an inserted domain that is proteolytically removed by furin-like proprotein convertases in the trans-Golgi network (19, 29)., resulting in the constitutive secretion of the mature enzyme into the extracellular matrix. This structural divergence dictates not only subcellular localization but also oligomeric behavior, as meprin α forms exceptionally large megadalton-scale oligomers, whereas meprin β predominantly exists as membrane-bound dimers (30). These biosynthetic and structural differences are mirrored by a highly ordered expression pattern within the epidermis. Meprin α is predominantly expressed in basal keratinocytes of the stratum basale. Furthermore, meprin α supports proliferative and matrix-associated processes without inducing cytotoxicity, consistent with its extracellular localization and oligomeric restraint. Conversely, meprin β expression is restricted to the suprabasal layers, particularly the stratum granulosum (19, 30).

**Figure 1:**
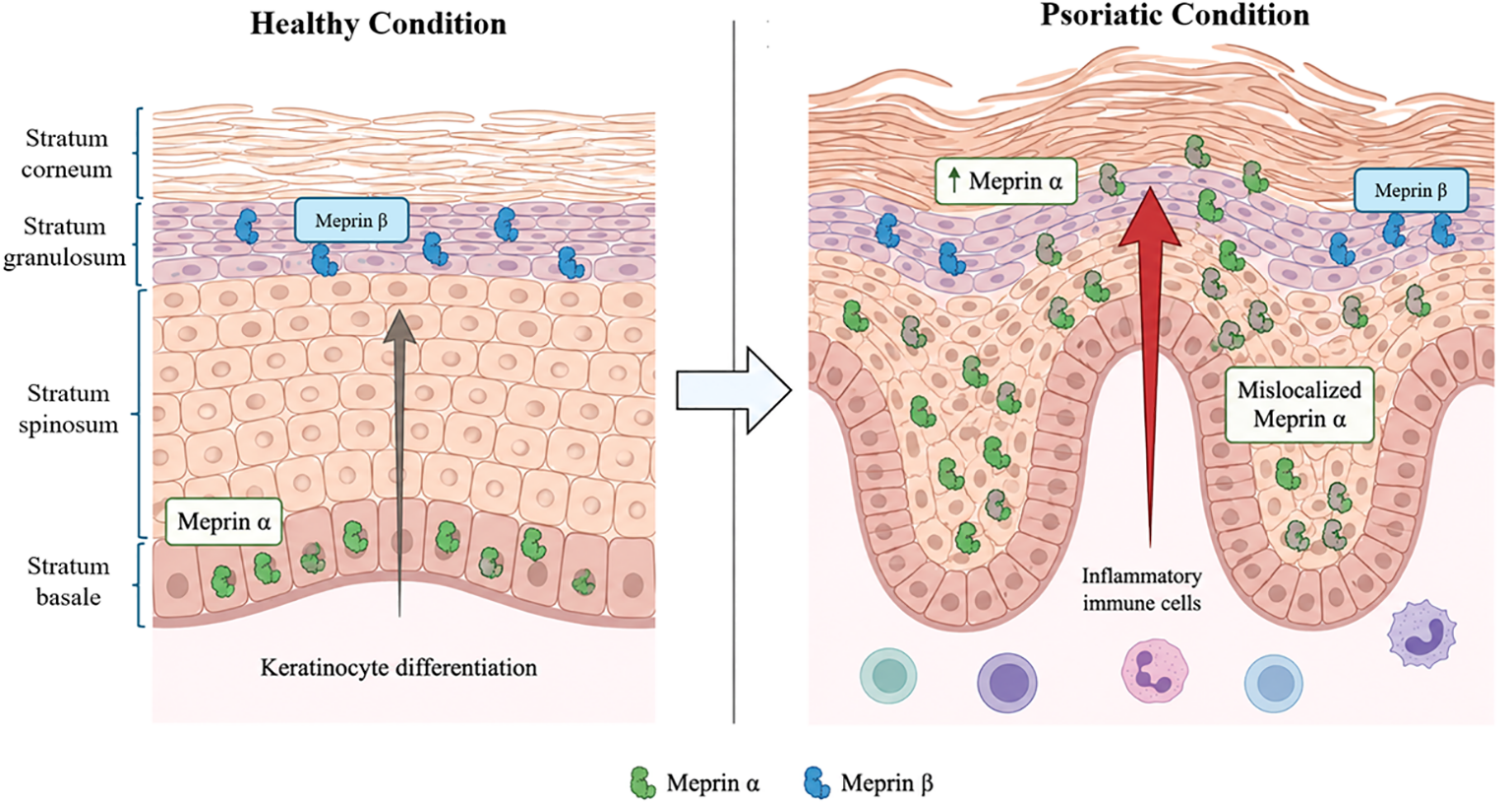
Physiological localization and pathological redistribution of meprin proteases in healthy and psoriatic epidermis. In healthy skin, meprin α is predominantly localized to basal keratinocytes of the stratum basale, whereas meprin β is mainly distributed within suprabasal and granular epidermal layers. This restricted expression pattern supports normal keratinocyte differentiation and epidermal homeostasis. In psoriatic skin, the epidermis exhibits characteristic hyperplasia and hyperkeratosis accompanied by increased meprin α expression and its pathological redistribution from the basal layer into suprabasal epidermal compartments. The altered localization pattern is associated with enhanced proteolytic activity and occurs within an inflammatory microenvironment characterized by immune-cell infiltration. The image highlights the transition from tightly regulated meprin localization in healthy epidermis to dysregulated meprin α expression and distribution in psoriasis. (Created using BioRender.com and assembled in Microsoft PowerPoint).

Functional studies in human keratinocytes have demonstrated that meprin β induces pronounced morphological changes and reduces cell viability (36)., linking its membrane-anchored proteolytic activity to late-stage differentiation and programmed cell death. Together, the mutually exclusive epidermal distribution of meprin α and β establishes a proteolytic gradient aligned with keratinocyte maturation, underscoring how structural organization directly translates into tissue-level function. The disruption of the physiological distribution of meprin isoforms in psoriatic skin highlights the significance of this spatial arrangement. Meprin α and meprin β show a highly organized and mostly non-overlapping expression pattern in healthy epidermis, with meprin α mostly found in basal keratinocytes and meprin β concentrated in the differentiated suprabasal compartments (19, 30). By limiting proteolytic activity to specific keratinocyte maturation phases, this compartmentalization helps preserve epidermal homeostasis. On the other hand, meprin α is significantly upregulated in psoriatic lesions and is redistributed from the basal layer to the more superficial epidermal compartments (19). Meprin α is exposed to a new substrate repertoire and inflammatory milieu due to its mislocalization, which may enhance the proteolytic processing of ECM proteins, cytokines, and differentiation-associated chemicals (**Figure 1**). This segregation functionally aligns meprin activity with discrete stages of epidermal differentiation. Meprin α supports terminal differentiation by processing extracellular matrix components and activating kallikrein-related peptidases (KLK)- dependent desquamatory pathways, thereby facilitating cornified envelope formation and controlled corneocyte shedding. In contrast, membrane-anchored meprin β promotes remodeling of cell-to-cell adhesion complexes and cleavage of membrane-associated substrates (19), including E-cadherin and pro-IL-1β (34, 36), thereby influencing the balance among proliferation, differentiation, and apoptosis during upward migration. The resulting proteolytic gradient establishes a maturation-dependent shift from proliferative signaling to terminal differentiation and barrier execution, ensuring coordinated epidermal stratification and homeostasis. The proteolytic activity of meprins is further regulated by zymogen activation. In the epidermal microenvironment, kallikrein-related peptidases (KLKs) act as physiological activators: KLK-5 activates both meprin α and β, whereas KLK-4 and KLK-8 preferentially activate meprin α. Activated meprins subsequently engage in reciprocal protease cascades by processing downstream substrates, such as pro-KLK-7, thereby amplifying and coordinating epidermal proteolysis (16, 37).

Beyond the epidermis, meprin expression extends into the dermis, where meprin α and β contribute to ECM organization (21). In addition to their established roles in fibrillar collagen processing, meprins contribute to the maturation of collagen VII, a key component of anchoring fibrils at the dermal–epidermal junction. Meprin-dependent processing of collagen VII facilitates basement membrane organization and epidermal adhesion, highlighting that these enzymes participate not only in ECM turnover but also in maintenance of skin architecture. This function is particularly relevant to psoriasis, where alterations in dermal–epidermal integrity and extracellular matrix remodeling are characteristic pathological features (38). Structurally, both enzymes function as unique C-terminal (carboxy-terminal) and N-terminal (amino-terminal) procollagen proteinases that cleave terminal propeptides from fibrillar collagens, distinguishing them from classical MMPs (33). Genetic ablation of either meprin subunit results in defective collagen fibrillogenesis and reduced dermal tensile strength (33, 39), highlighting the vital roles of these subunits in maintaining skin integrity. Dysregulated collagen remodeling is a recognized hallmark of psoriatic skin, characterized by epidermal hyperplasia, altered ECM composition, neovascularization, and inflammatory leukocyte infiltration. The observed upregulation and aberrant epidermal localization of meprin α in psoriatic lesions further suggest that meprin-dependent proteolytic networks may directly contribute to these structural and inflammatory alterations. Given that meprin α and β directly regulate procollagen maturation and fibril assembly, aberrant expression or activity may contribute to the disorganized collagen architecture and reduced mechanical stability observed in psoriasis. Moreover, meprin-mediated processing of pro-inflammatory mediators, such as IL-1β, provides an additional mechanistic link between ECM remodeling and sustained cutaneous inflammation (40)., suggesting that altered meprin activity may integrate structural and inflammatory pathways in psoriatic pathology.

Collectively, the integration of multidomain structures, regulated biogenesis, and spatially restricted expression positions meprins as key nodes within cutaneous protease networks, rather than passive degradative enzymes. Consequently, alterations in meprin abundance or localization, as observed in psoriatic lesions, may profoundly influence inflammatory signaling, tissue remodeling, and barrier integrity. These observations have prompted increasing interest in the pathological roles of meprins in inflammatory skin disorders (**Table 2**).

**Table 2:** Comparative functional properties of Meprin α and Meprin β.

Feature	Meprin-α	Meprin-β	Functional Implications
Gene	MEP1A	MEP1B	Distinct genetic control under inflammatory signals
Subcellular localization	Secreted extracellularly after furin cleavage	Membrane bound, may shed by ADAM proteases	Determines substrate accessibility and local proteolysis
Oligomerization	Forms large soluble oligomers	Exists as dimers or α/β heterodimers	Influences enzyme stability and diffusion range
Activation mechanism	Activated by KLK-4, KLK-5, KLK-8	Activated by KLK-5, trypsin like proteases	Links to epidermal protease cascades
Major substrates	ECM proteins (collagen, laminin, nidogen), cytokines	IL-1β, IL-18, IL-6R, E-cadherin, syndecan-1, APP	Shapes inflammation, cell adhesion and tissue architecture
Tissue distribution	Basal keratinocytes, intestine, kidney	Suprabasal keratinocytes, kidney, immune epithelia	Layer specific functions in epidermis
Regulation	Upregulated by inflammatory cytokines (IL-17, TNF-α)	Upregulated by TGF-β, IL-1β	Responsive to chronic inflammatory cues
Inhibition	Fetuin A, Cystatin C	Fetuin A, Cystatin C	Endogenous inhibitors maintain proteolytic balance
Pathogenic role in psoriasis	Promotes aberrant keratinocyte differentiation, ECM degradation	Activates cytokines and receptor shedding leading to sustained inflammation	Both isoforms contribute to chronic inflammation and barrier loss

## 3 Pathological implications of meprins

Building on the structural and physiological framework discussed above, this section examines the growing body of evidence that positions meprins as central modulators of pathological remodeling, inflammation, and tumorigenesis (5, 41, 42). Although meprins were initially characterized as epithelial proteases with localized physiological functions (18, 19). Emerging evidence has positioned them as multifaceted mediators of pathology in diverse inflammatory, fibrotic, and neoplastic contexts (43, 44). In pathological conditions characterized by tissue remodeling, barrier failure, and chronic inflammation, meprin expression and activity are increased. Elevated levels of meprin α and β have been linked to cancer, where they facilitate extracellular matrix remodeling, cytokine activation, and tumor growth, as well as inflammatory and fibrotic illnesses such as systemic sclerosis, renal damage, and inflammatory bowel disease. In the skin, meprins are induced under inflammatory and barrier-disrupted conditions, with psoriatic lesions exhibiting both increased expression and aberrant localization of meprin α. These alterations have been linked to enhanced keratinocyte proliferation, disturbed epidermal differentiation, and amplification of inflammatory signaling pathways, suggesting that dysregulated meprin activity may actively contribute to disease pathogenesis rather than merely reflect ongoing tissue inflammation. Importantly, their expression is not restricted to psoriasis, but has also been reported in other cutaneous disorders, including Netherton syndrome, atopic dermatitis, and keloid-associated inflammation, highlighting a broader role in skin pathology (45–47).

**Figure 2:**
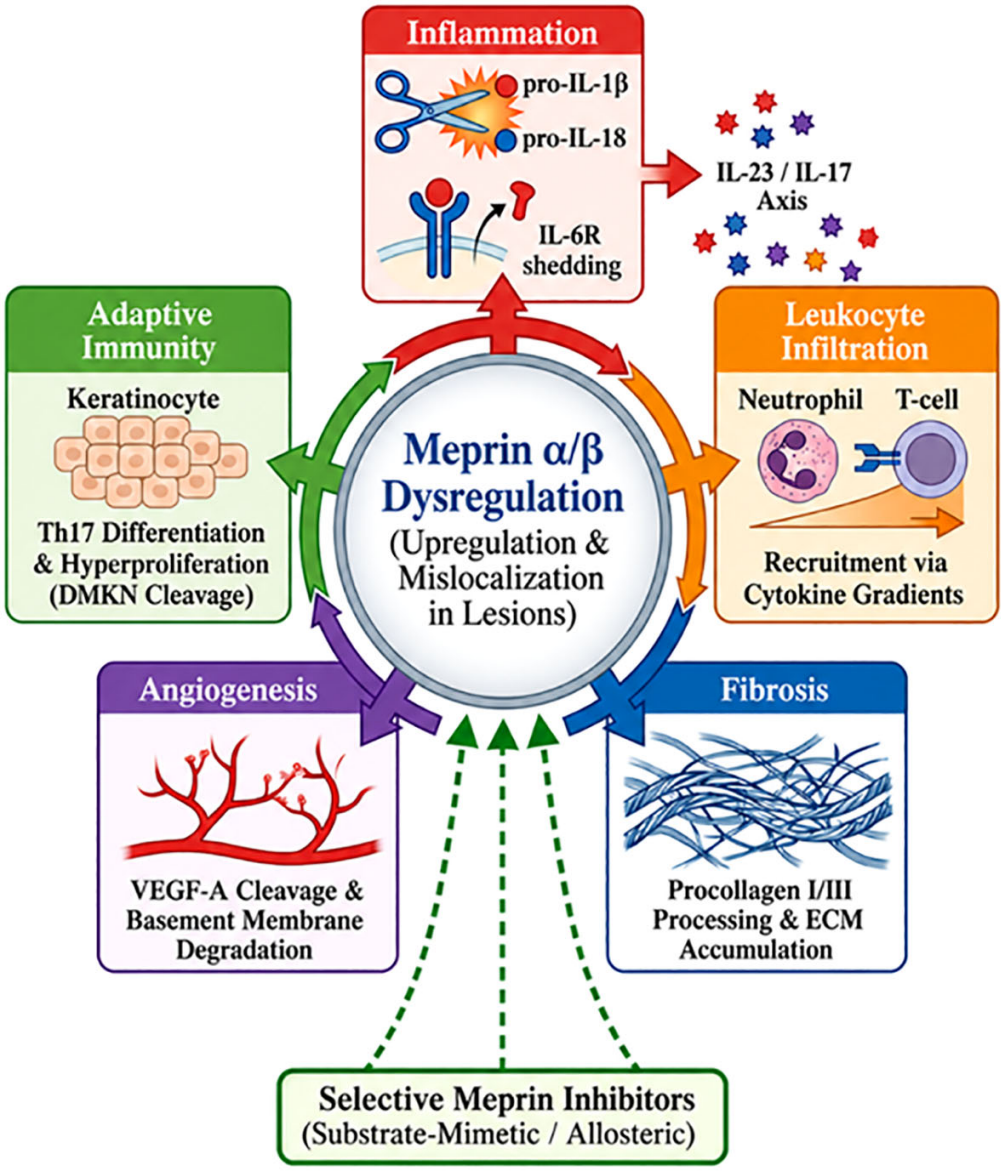
Meprin α/β dysregulation links extracellular proteolysis to psoriatic inflammation and barrier dysfunction. Abnormal expression and mislocalization of meprin α and β may enhance shedding of IL-1β, IL-18, and IL-6R, as well as IL-23/IL-17-driven leukocyte infiltration in psoriasis. Dysregulated proteolysis promotes keratinocyte hyperproliferation, fibrosis via procollagen processing, and angiogenesis through VEGF-A cleavage, compromising barrier integrity and increasing TEWL. DMKN, Dermokine; IL, Interleukin; TEWL, Transepidermal Water Loss; VEGF-A, Vascular Endothelial Growth Factor-A. (Created using BioRender.com and assembled using Microsoft PowerPoint).

Their unique substrate repertoire, which includes cytokines, adhesion molecules, and ECM proteins, enables them to influence processes such as inflammation, immune cell infiltration, and tissue remodeling (19, 33, 34, 48). Dysregulated meprin activity has been implicated in sustaining chronic inflammatory loops, compromising barrier integrity, and altering angiogenic signaling. Unlike canonical intracellular proteases, meprins act extracellularly, bypassing traditional regulatory checkpoints and exerting long-range effects within the tissue microenvironment. Consequently, aberrant expression or activation of meprins may convert physiological remodeling into persistent pathology, linking extracellular proteolysis to the initiation and progression of chronic inflammatory diseases, such as psoriasis (**Figure 2**).

### 3.1 Inflammation

Meprins have emerged as critical modulators of inflammatory signaling through selective proteolytic control of cytokines and associated receptors (23, 28). They exert highly specific effects on inflammatory cascades by reshaping cytokine bioavailability, signal duration, and spatial range. Aberrant regulation of these proteases is associated with persistent and dysregulated inflammatory responses. The primary inflammatory function of meprins is their capacity to regulate members of the IL-1 cytokine family. Meprin β directly processes pro-IL-18 into its biologically active form independently of canonical inflammasome-mediated caspase-1 cleavage (49). This non-canonical activation pathway enables sustained IL-18 signaling in inflammatory microenvironments, where intracellular inflammasome activation is either limited or bypassed. Studies have shown that loss of meprin β activity leads to reduced IL-18 maturation and reduced inflammatory severity, indicating that meprin β is a key extracellular amplifier of IL-18-driven inflammation (19, 49).

In contrast to inflammasome-restricted cytokine activation, meprin-dependent IL-18 processing occurs extracellularly (31, 49) and is therefore less tightly regulated. This spatial uncoupling of cytokine maturation from immune cell activation checkpoints contributes to prolonged IFN-γ production (50), enhanced immune cell activation, and sustained inflammatory signaling. These mechanisms are particularly relevant in chronic inflammatory disorders, where IL-18 plays a pathogenic role. In psoriasis, elevated IL-18 levels correlate with disease severity (51, 52)and contribute to Th1 polarization and sustained IFN-γ production within the lesional skin (51). Therefore, extracellular activation of IL-18 by meprin β may potentiate pathogenic cytokine loops independent of inflammasome regulation, amplifying chronic inflammation in psoriatic microenvironments. Beyond IL-18, meprins also influence inflammatory signaling by regulating IL-6 pathways at multiple levels. Meprin α and meprin β both function as IL-6 receptor sheddases, cleaving membrane-bound IL-6R to generate soluble IL-6R (53). This promotes IL-6 trans-signaling, which preferentially activates pro-inflammatory pathways and expands IL-6 responsiveness in cells lacking membrane-bound IL-6R expression. Meprin-mediated IL-6R shedding shifts IL-6 signaling away from homeostatic regulation toward sustained inflammatory activation. Given that IL-6 and IL-6 trans-signaling promote keratinocyte hyperproliferation and inflammatory cell recruitment in psoriasis, meprin-dependent IL-6R shedding may further exacerbate epidermal thickening and immune activation characteristic of the disease (48, 54, 55).

**Figure 3:**
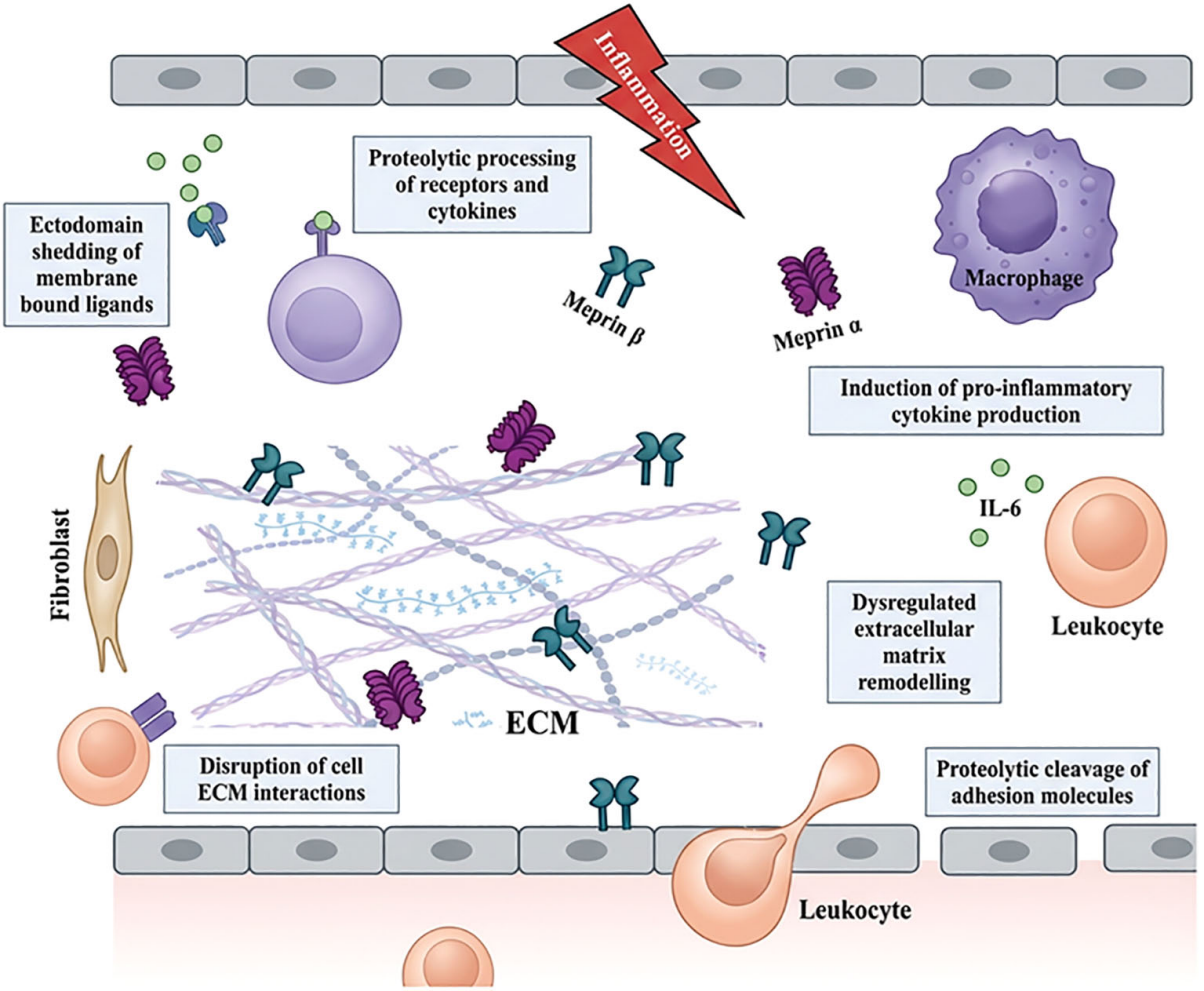
Meprin-mediated extracellular matrix remodeling and inflammatory amplification in psoriasis. Meprin α and meprin β contribute to psoriatic pathogenesis through the proteolytic processing of cytokines, cell-surface substrates, and extracellular matrix (ECM) components. Meprin-mediated cytokine activation promotes the production of pro-inflammatory mediators, including IL-6, thereby enhancing immune-cell recruitment and sustaining inflammatory signaling. In parallel, cleavage of adhesion molecules and ECM remodeling disrupt cell–ECM interactions, facilitating leukocyte migration and tissue infiltration. Interactions between meprins, stromal cells, and immune cells collectively contribute to the establishment of a psoriatic inflammatory microenvironment characterized by chronic inflammation and ongoing tissue remodeling. ECM, extracellular matrix; IL-6, interleukin-6. (Created using BioRender.com and assembled using Microsoft PowerPoint).

The inflammatory consequences of IL-6R shedding depend on meprin localization. Membrane-bound meprin β exhibits robust IL-6R sheddase activity, whereas soluble meprin β displays markedly reduced capacity to process IL-6R (48, 54, 56). This highlights how inflammatory outcomes are governed by protease expression levels, as well as by post-translational regulation and cellular compartmentalization of meprins. Meprins further modulate inflammation by directly processing IL-6. The limited cleavage of IL-6 by meprin α and meprin β reduces cytokine activity by disrupting receptor engagement. Elevated IL-6 levels observed in *in vivo* meprin-deficient models suggest that meprins contribute to cytokine turnover and termination of inflammatory signaling (55). These observations emphasize the dualistic nature of meprins: while promoting IL-6 trans-signaling via receptor shedding, meprins simultaneously restrain excessive IL-6 accumulation by inactivating the cytokine. Hence, the net inflammatory outcome reflects the balance between these competing proteolytic events. At the level of immune cell regulation, meprins indirectly contribute to inflammatory amplification by modulating cytokine availability, rather than acting directly on immune cells (53). Meprins are not classical immune cell proteases; rather, they regulate the extracellular inflammatory milieu to which immune cells respond. This enables meprins to influence leukocyte activation thresholds, cytokine gradients, and inflammatory persistence without directly engaging the immune cell signaling machinery. By enabling extracellular cytokine maturation and receptor shedding, meprins amplify inflammatory signals while bypassing the tightly regulated immune checkpoints. Isoform-specific activity, spatial restriction, and proteolytic selectivity further define the function of meprins as amplifiers or attenuators of inflammation (**Figure 3**).

### 3.2 Leukocyte infiltration

Chemokine gradients, cytokine activation, engagement of adhesion molecules, and local proteolytic remodeling of the inflammatory milieu are all part of the tightly controlled, multistep process of leukocyte recruitment to inflammatory tissues (57, 58). Meprins are underestimated but physiologically significant modulators of leukocyte infiltration during inflammation, even though MMP and ADAM proteases have traditionally dominated discussions of protease-mediated immune modulation (16, 19, 28). Importantly, the contribution of meprins extends beyond structural tissue remodeling and reflects their capacity to modulate inflammatory signaling at the levels of cytokine activation, chemokine availability, and leukocyte responsiveness. Although they are most abundantly expressed in the proximal renal tubules, meprins are also detected in monocytes, macrophages, and discrete leukocyte populations, suggesting direct participation in immune processes rather than passive activity.

Meprins preferentially process pro-inflammatory cytokines, which is a key mechanism by which they affect leukocyte infiltration. Meprin β has been shown to activate pro-IL-1β and pro-IL-18 by proteolytic cleavage, generating bioactive cytokines independent of canonical inflammasome-mediated cleavage. In contrast, meprin-mediated processing of IL-6 results in cytokine inactivation, underlining the dual and context-dependent nature of meprin activity. This substrate specificity is relevant to leukocyte recruitment, as IL-1β and IL-18 are potent drivers of chemokine expression, endothelial activation, and neutrophil and T cell infiltration, whereas IL-6 signaling exerts more pleiotropic and, in some contexts, regulatory effects (19, 51–55). The pathological relevance of this proteolytic balance is illustrated by studies employing the genetic ablation of meprin β in inflammatory disease models. In a unilateral ureteral obstruction (UUO)-induced renal inflammation mouse model, meprin β-deficient C57BL/6 mice exhibited reduced leukocyte accumulation in obstructed kidneys compared with wild-type mice. Flow cytometry revealed a significant decrease in Ly6G^+^ neutrophils and CD3^+^ T cells, indicating that meprin β promotes infiltration of both innate and adaptive immune cells during inflammation (59). Interestingly, these effects were unchanged in the baseline leukocyte populations, suggesting that meprin β has a function in inflammation-induced recruitment.

This suggests that meprin β amplifies inflammatory leukocyte infiltration by enhancing the local availability of active cytokines that direct immune cell recruitment. Beyond cytokine activation, meprins may influence leukocyte recruitment by modulating adhesion molecule expression and chemokine bioactivity. Meprin β has been shown to cleave E-cadherin and other cell-surface proteins, potentially altering epithelial barrier integrity and facilitating transendothelial migration. Additionally, proteolytic processing of extracellular matrix components by meprins can expose cryptic binding sites and generate matrikines that regulate leukocyte chemotaxis (34). Emerging evidence suggests that meprin-dependent cleavage of cytokine receptors may modulate endothelial responsiveness, thereby indirectly influencing leukocyte rolling, adhesion, and extravasation during inflammatory responses (35). The observed reduction in neutrophil and T-cell accumulation in meprin β-deficient kidneys is consistent with impaired activation of IL-1β and IL-18, both of which are critical for maintaining chemokine gradients and endothelial adhesion molecule expression during inflammation (49, 51, 52). This pathway provides an alternative route for cytokine activation that bypasses inflammasome dependency, explaining inflammatory infiltration in contexts where canonical inflammasome signaling is attenuated or spatially restricted. Complementary insights arise from endotoxemia and LPS-driven inflammation in mouse models examining the role of meprin α complexes. C57BL/6 mice with meprin α deficiency displayed attenuated leukocyte infiltration in both renal and bladder inflammation following LPS injection. Reduced myeloperoxidase activity, diminished tissue edema, and lower accumulation of inflammatory cells in meprin α-deficient mice indicate that meprin α contributes to leukocyte recruitment during inflammation (60). These effects are accompanied by altered cytokine kinetics, including reduced, more rapidly resolving levels of TNF-α and IL-1β, suggesting that meprin activity shapes not only local but also systemic inflammatory signaling. *In vitro* studies have demonstrated that meprin deficiency impairs leukocyte migratory capacity, supporting a cell-intrinsic role for meprin. *In vivo*, specialized regulatory activities, rather than widespread immunosuppression, are supported by the selective reduction of specific leukocyte subsets (27, 35, 61).

Thus, sustained or dysregulated meprin activity may exacerbate inflammatory tissue damage by promoting excessive leukocyte infiltration. The ability of meprin proteins to inactivate IL-6 and certain chemokines suggests that they may also play anti-inflammatory or resolution-promoting roles in specific inflammatory milieus. Thus, the impact of meprin activity on leukocyte infiltration is likely dictated by the local cytokine landscape, protease expression, and inflammation dynamics. In psoriasis, excessive leukocyte infiltration into both the dermis and epidermis is a defining histopathological feature characterized by neutrophil accumulation (Munro microabscesses), dermal dendritic cell activation, and Th1/Th17 cell expansion (62, 63). IL-1β and IL-18 contribute to chemokine production (e.g., CXCL1 and CXCL8) and endothelial activation, thereby sustaining immune cell recruitment to the psoriatic plaques. Given the capacity of meprin β to activate IL-1β and IL-18 extracellularly, aberrant meprin activity may potentiate leukocyte influx in psoriatic skin by amplifying cytokine-driven chemokine gradients, independent of inflammasome restriction (60, 64). Conversely, context-dependent IL-6 inactivation by meprins may influence the balance between chronic inflammatory persistence and resolution, suggesting that meprins can modulate both the intensity and duration of leukocyte infiltration in psoriasis.

### 3.3 Angiogenesis

Angiogenesis results from the convergence of proteolytic ECM remodeling, growth factor bioavailability, and inflammatory signaling (65, 66). Meprins occupy a distinct niche within this combination, functioning at the interface of matrix processing and cytokine regulation (5, 15). Local disruption of the basement membrane is a major prerequisite for angiogenesis, facilitating endothelial migration and sprouting. Meprins directly cleave structural components of the basement membrane, including collagen IV, laminins, and nidogen, thereby weakening endothelial anchorage and altering the biomechanical cues governing vessel stability. Mislocalized or high meprin activity promotes premature basement membrane dissolution, resulting in immature and dysfunctional neovessels (67). In diseased conditions, sustained meprin-mediated proteolysis shifts this balance toward uncontrolled endothelial invasion and defective vessel maturation. In addition to structural remodeling, meprins regulate angiogenic signaling by proteolytic processing of growth factors and their extracellular reservoirs. Meprins cleave vascular endothelial growth factor-A (VEGF-A), the main regulator of angiogenesis, to generate fragments that retain angiogenic activity but exhibit altered receptor engagement (68). Such processing not only amplifies VEGF availability but also reshapes the signaling output, potentially biasing endothelial responses toward proliferation rather than stabilization. This forms a conceptual framework for the observation that meprin-rich microenvironments support exuberant yet disorganized vascular growth, a hallmark of tumor-associated angiogenesis (65, 67, 69–71).

The relevance of meprin-driven angiogenesis is evident in cancer, where meprin α expression and activity are elevated at invasive tumor fronts and within the stromal compartments. Secreted meprin α accumulates in the tumor microenvironment, extending its proteolytic reach beyond tumor cells to adjacent endothelial and stromal cells (16, 41). This extracellular localization ensures the coordinated modulation of ECM architecture and angiogenic factor gradients, facilitating both neovascularization and tumor cell dissemination. Additional context for the role of meprin is provided by inflammation-associated angiogenesis. Inflammatory tissues are characterized by persistent cytokine signaling and ongoing vascular remodeling. Meprins process multiple cytokines and membrane-bound ligands implicated in inflammatory angiogenesis, including interleukin family members and epidermal growth factor receptor ligands. Meprins amplify inflammatory angiogenic coupling and sustain endothelial activation and leukocyte recruitment via these cytokines. Meprin β, locally regulates endothelial cell junctions and pericellular proteolysis, whereas soluble meprin α exerts broad-spectrum effects within the extracellular space. Therefore, the comparative abundance, activation state, and inhibition of each isoform determine angiogenic outcomes (15, 16, 32, 35, 67, 71).

Inhibitors such as fetuin A and cystatin C impose an additional regulatory layer that appears to be disrupted in disease states, leading to unregulated proteolytic activity. Both fetuin and cystatin act as endogenous brakes on extracellular proteolysis, thereby maintaining protease activity. Fetuin A, a circulating glycoprotein synthesized by hepatocytes, acts as an inhibitor of metalloprotease activity by directly binding proteolytic enzymes and modulating their accessibility to extracellular substrates, thereby limiting excessive degradation of ECM components and growth factor reservoirs and ensuring matrix integrity. Cystatin C, an inhibitor of cysteine proteases, constrains metalloprotease-driven angiogenic remodeling by restricting protease cross-activation cascades within the perivascular microenvironment, thereby fine-tuning matrix turnover, endothelial cell migration, and lumen formation by suppressing proteolytic amplification (72). Disruption of these inhibitors skews the regulatory balance toward excessive proteolysis, resulting in aberrant ECM degradation, dysregulated release of proangiogenic mediators, and unstable neovessels (15, 16). This imbalance is recognized as a contributing factor to pathological angiogenesis in chronic inflammation and vascular disease.

Dysregulated meprin activity reshapes angiogenic niches by simultaneously altering ECM architecture, growth factor signaling, and inflammatory crosstalk. Rather than acting as generic matrix-degrading enzymes, meprins function as context-sensitive regulators of pathological angiogenesis, contributing to vascular dysfunction and chronic inflammatory diseases. In psoriasis, angiogenesis is an early and defining event, characterized by dilated, hyperpermeable dermal capillaries that sustain immune cell recruitment and promote epidermal hyperplasia. Elevated VEGF-A levels in psoriatic lesions correlate with disease severity, underscoring the contribution of pathological vascular remodeling to plaque persistence. Given the capacity of meprins to process VEGF-A and remodel basement membrane components, dysregulated meprin activity may further destabilize the dermal microvasculature, thereby reinforcing the inflammatory angiogenic feedback loop in psoriatic skin (66, 69, 70, 73).

### 3.4 Skin barrier integrity

The skin barrier is a specialized, multilayered structure that protects the host from external cues and prevents excessive transepidermal water loss (TEWL) (74). Coordinated keratinocyte development, junctional assembly, and controlled proteolysis are necessary for maintaining barrier integrity. As a result, meprins are important regulators of epidermal homeostasis, and inflammatory and hyperproliferative skin conditions are exacerbated by their dysregulation (26, 74, 75). Meprins were initially characterized as epithelial metalloproteases abundantly expressed in the brush-border membranes of proximal renal tubules and intestinal epithelial cells, where they contribute to extracellular matrix turnover, epithelial differentiation, and barrier maintenance (25, 76). These early studies established meprins as tissue-restricted regulators of epithelial homeostasis long before their involvement in inflammatory skin diseases was recognized.

With secreted meprin α localized to basal keratinocytes and membrane-bound meprin β to suprabasal layers, meprin isoforms play spatially separate but complementary functions in the epidermal structure, enabling layer-specific regulation of differentiation and barrier function. Meprins function in tandem with structural proteins and KLK-mediated desquamation to regulate cytokine activation and junctional integrity within the larger protease network. Meprin α is increased and mislocalized in pathological conditions, including psoriasis, thereby disrupting protease balance, weakening barrier cohesion, and promoting aberrant keratinocyte growth (15, 34, 37, 45, 77). Experimental induction of epidermal meprin α overexpression in C57BL/6 transgenic mouse models recapitulates the key hallmarks of inflammatory skin diseases, including acanthosis, hyperkeratosis, parakeratosis, and increased TEWL (24). These findings demonstrate that dysregulated meprin α expression is sufficient to initiate barrier dysfunction independent of primary immune activation, suggesting that protease imbalance may function as an upstream trigger rather than merely a downstream consequence of inflammation. Proteomic and N-terminomics studies (an approach that identifies newly generated protein N-termini to map protease cleavage events) suggest an altered epidermal degradome, with substrates implicated in cytoskeletal organization, cell adhesion, and stress responses being preferentially cleaved due to the overexpression of meprin α (2). Meprin α-mediated cleavage of dermokine (DMKN), a keratinocyte-derived protein implicated in epidermal differentiation and immune modulation, leads to DMKN deficiency, resulting in keratinocyte hyperproliferation and increased leukocyte recruitment. These findings position meprin α not only as a downstream effector but also as an upstream driver of pathological signaling cascades that destabilize the skin barrier. The identification of dermokine (DMKN) as a direct substrate further supports a model in which meprin α selectively targets differentiation-associated proteins to reprogram keratinocyte behavior (2, 15, 24, 77). Loss of DMKN function is associated with heightened inflammatory susceptibility, indicating that meprin-mediated DMKN depletion may couple structural barrier defects with immune activation, thereby reinforcing a feed-forward inflammatory loop (78, 79). Meprin β, on the other hand, contributes to barrier regulation through partially distinct mechanisms. As a membrane-bound sheddase, it directly regulates cell-to-cell and cell-to-matrix interactions by cleaving adhesion molecules and proteoglycans (18). Furthermore, in the epidermis, meprin β sheds syndecan-1, which is vital for maintaining keratinocyte cohesion and growth factor gradient. Dysregulation of syndecan-1 shedding disrupts terminal differentiation and alters epidermal architecture. Because syndecan-1 regulates growth factor gradients and keratinocyte adhesion, excessive shedding may perturb spatial signaling cues that normally restrict proliferation to the basal layers (80). This suggests that meprin β-mediated ectodomain shedding does not merely alter structural adhesion but may reconfigure epidermal growth factor availability, thereby contributing to the hyperproliferative phenotypes observed in inflammatory skin diseases.

Meprin action encompasses not only substrate cleavage but also the localization-dependent modulation of epidermal gene expression. Heterodimerization with membrane-bound meprin β secures meprin α at the cell surface, thereby enhancing its accessibility to substrates involved in barrier integrity and immune signaling. Loss-of-function mouse models demonstrate compromised wound healing and extracellular matrix architecture, whereas increased meprin expression in hyperkeratotic skin conditions underscores the detrimental effects of excessive proteolysis (14–16, 29). Collectively, meprins function as nodal regulators that connect proteolysis, epithelial development, and immunological signaling, integrating barrier integrity with inflammatory responses. Rather than being overactive, their pathogenic effects stem from geographical mislocalization and disruption of coordinated protease networks. This establishes meprin as a key yet challenging therapeutic target at the interface of barrier biology and immunity.

## 4 A mechanistic framework for meprins’ involvement in psoriasis: a perspective

Psoriasis is an inflammatory disease in which adaptive immune responses, particularly those driven by Th17 and Th1 cells, are sustained by signaling between keratinocytes and immune cells within the epidermal niche (1). Dysregulated extracellular proteolysis is a critical yet underexplored regulator of the immune-epithelial interface by modulating cytokine activation, antigen availability, and tissue architecture. Meprins are expressed in the human epidermis in a differentiation-dependent manner, with meprin α and β localized within basal keratinocytes and in suprabasal layers, respectively, under normal circumstances. This expression pattern is altered in hyperproliferative epidermal conditions, such as psoriasis, indicating dysregulated proteolytic activity and disrupted keratinocyte differentiation. Keratinocytes play an active role in adaptive immunological activation via cytokine release and antigen presentation; hence, spatial redistribution is mechanistically significant (14–16, 19, 77, 81).

The IL-23–IL-17 axis is exaggerated in psoriasis, thereby promoting Th17 cell expansion and enhancing keratinocyte activation. Meprin α and β cleave pro-IL-1β into its active form independently of inflammasome-mediated caspase-1 activation (61). Meprin β processes pro-IL-18, generating mature IL-18 that enhances Th1 and Th17 polarization (82). Extracellular activation of meprins initiates a parallel proteolytic pathway that sustains adaptive immune responses in psoriatic lesions, with IL-1β and IL-18 serving as vital signals for adaptive T cell differentiation. Keratinocyte-derived cytokines are key drivers of immune cell recruitment and persistence in psoriasis, and their extracellular processing profoundly influences the strength and duration of signaling. By operating outside the cell and independently of canonical inflammasome pathways, meprins might extend inflammatory signaling beyond the temporal constraints of intracellular cytokine maturation (9, 14, 17, 19, 64, 83,).

In addition to cytokine maturation, meprin activity may directly alter keratinocyte fate by selectively processing epidermal substrates. Recent evidence identified dermokine (DMKN), a keratinocyte-derived regulator of epidermal differentiation and immune homeostasis, as a direct substrate of meprin α. In a transgenic mouse model, pathological overexpression of meprin α led to extensive degradation of DMKN. This overexpression induced a psoriasis-like phenotype characterized by epidermal hyperplasia, hyperkeratosis, increased transepidermal water loss, and enhanced leukocyte infiltration. These findings suggest that the meprin α–DMKN axis represents a direct mechanistic link between dysregulated extracellular proteolysis and epidermal dysfunction. Importantly, degradomic analyses further demonstrated preferential cleavage of proteins involved in cytoskeletal organization, cell adhesion, and stress-response pathways, indicating that aberrant meprin α activity directly degrades structural networks required for keratinocyte cohesion, cytoskeletal integrity, and epidermal barrier stability. In parallel, meprin β cleaves adhesion molecules such as E-cadherin and promotes shedding of syndecan-1. Disruption of cytoskeletal and adhesion-associated proteins may have important consequences for epidermal organization. Loss of E-cadherin-mediated cell-cell contacts can impair keratinocyte cohesion, alter differentiation programs, and weaken barrier integrity. In contrast, degradation of cytoskeletal networks may compromise cellular architecture and mechanical stability. Together, these changes provide a plausible mechanism linking aberrant meprin activity to epidermal remodeling and persistent inflammation in psoriasis. Collectively, these proteolytic events provide a basis for how dysregulated meprin activity can simultaneously compromise barrier integrity, facilitate leukocyte infiltration, and amplify inflammatory signaling in psoriatic skin.in psoriatic skin.

Large-scale degradomic approaches strengthen the mechanistic significance of these findings. Terminal amine isotopic labeling of substrates (TAILS) and related N-terminomic methodologies have identified numerous extracellular matrix proteins, adhesion molecules, cytokine regulators, and epidermal proteins as direct or indirect meprin substrates. Rather than acting on isolated targets, these studies indicate that meprins participate in a broader epidermal degradome network that can simultaneously influence tissue architecture, barrier integrity, and inflammatory signaling. Such systems-level analyses provide important experimental support for the concept that dysregulated meprin activity can reshape the cutaneous microenvironment through coordinated substrate processing (23).

Importantly, the relevance of these mechanisms is reinforced by complementary genetic mouse models that together define the contribution of meprins to inflammatory and epidermal pathology. Loss-of-function studies demonstrate that meprin activity is required for the full development of inflammatory responses: Mep1α-deficient mice exhibit attenuated inflammatory cell infiltration and reduced tissue injury, whereas Mep1β-deficient mice impair IL-18 maturation and diminish leukocyte recruitment in inflammatory settings (82, 84). Conversely, gain-of-function studies provide evidence that excessive meprin activity is sufficient to induce pathological skin remodeling. Keratinocyte-specific overexpression of meprin α (K5Mα mice) results in epidermal hyperplasia, hyperkeratosis, increased transepidermal water loss, inflammatory cell infiltration, and degradation of dermokine, culminating in a psoriasis-like phenotype (24). Collectively, these complementary loss- and gain-of-function models provide convergent *in vivo* evidence that dysregulated meprin activity is not just associated with psoriatic pathology but can actively drive inflammatory amplification, barrier dysfunction, and epidermal remodeling. Together, these models establish a mechanistic continuum wherein physiological meprin activity contributes to epidermal homeostasis. In contrast, aberrant expression or activity shifts the cutaneous proteolytic environment toward chronic inflammation and psoriasiform disease.

Concurrently, both meprin α and meprin β function as interleukin-6 receptor (IL-6R) sheddases, generating soluble IL-6R and thereby facilitating IL-6 trans-signaling (15, 23, 53). Given the established role of IL-6 trans-signaling in keratinocyte activation and chronic cutaneous inflammation, meprin-mediated IL-6R shedding may further amplify pathogenic immune–epithelial crosstalk in psoriatic lesions (55). Collectively, these observations support a model in which meprins simultaneously promote pro-inflammatory cytokine activation, impair epidermal structural integrity, and enhance the propagation of inflammatory signals, thereby reinforcing the self-sustaining pathogenic circuits that characterize psoriasis.

Beyond cytokine maturation, meprins regulate ECM composition by cleaving collagens, laminins, and proteoglycans, thereby modifying tissue stiffness and immune cell migration routes. Extracellular matrix (ECM) remodeling is a determinant of T cell positioning and dendritic cell activation in inflamed skin (13). Proteolytic ECM fragments generated by meprins can function as DAMPs, engaging pattern recognition receptors on antigen-presenting cells (APCs) and reinforcing adaptive immune activation (85, 86). Psoriatic epidermis exhibits enhanced antigen presentation and aberrant exposure to self-antigens, thereby sustaining autoreactive T-cell responses (87). Meprin-mediated cleavage of adhesion molecules and pericellular substrates may increase antigen accessibility by disrupting keratinocyte cohesion and barrier integrity. Such proteolytic remodeling provides a permissive environment for sustained adaptive immune engagement without necessitating overt tissue destruction. Although direct studies linking meprins to psoriasis-specific T-cell responses are limited, evidence from inflammatory models demonstrates that meprin activity regulates leukocyte migration through extracellular matrices. Adaptive immune infiltration into psoriatic skin depends on efficient T cell trafficking and retention within the epidermis. By modulating ECM density and chemokine gradients, meprins may indirectly shape the magnitude and persistence of adaptive immune infiltration (87).

Adaptive immune responses in psoriasis are further reinforced by transcriptional networks dominated by AP-1, STAT3, and NF-κB, which regulate cytokine expression and keratinocyte proliferation. Meprin expression is sensitive to inflammatory transcriptional cues and protease cascades activated during chronic inflammation. This reciprocal regulation suggests a feed-forward loop in which adaptive immune signaling enhances protease expression, which in turn sustains immune activation by processing extracellular substrates (88).

Meprin inhibitors, like fetuin A and cystatin C, constrain proteolytic activity under homeostatic conditions but are frequently dysregulated in chronic inflammatory diseases (72). Loss of this inhibitory balance may permit excessive extracellular cytokine activation and matrix remodeling, thereby reinforcing maladaptive immune responses in psoriasis. Such dysregulation is consistent with the emerging concept of the extracellular “protease web” as an integrated signaling network rather than a collection of isolated enzymes (37, 89). From a therapeutic perspective, current psoriasis treatments target downstream cytokines, such as IL-17 and IL-23, with remarkable clinical efficacy; however, these approaches do not address the upstream extracellular processes that sustain adaptive immune activation. Selective modulation of meprin activity could theoretically attenuate cytokine maturation and immune cell recruitment while preserving essential immune surveillance, although this remains experimentally unproven in psoriasis cases.

Together, meprin proteins represent plausible yet underappreciated regulators of adaptive immune responses in psoriasis by coordinating extracellular cytokine activation, matrix remodeling, and immune cell accessibility. While direct causal evidence remains limited, the integration of existing biochemical, epidermal, and immunological data supports the hypothesis that meprins contribute to the persistence and amplification of adaptive immune circuits characteristic of psoriatic disease. Elucidating the precise role of meprins in psoriasis may reveal new opportunities to intervene upstream of the established cytokine pathways.

## 5 Leveraging meprins as a therapeutic target in psoriasis

Current therapeutic strategies for psoriasis predominantly target cytokine signaling nodes, including TNF, IL-17, and IL-23, thereby validating immune pathways as central drivers of disease while leaving tissue-intrinsic amplification mechanisms comparatively unexplored (9). Extracellular proteolysis represents one such amplification layer, as proteases regulate cytokine activation, receptor availability, and ECM architecture in inflamed skin. Meprins cleave substrates implicated in inflammatory signaling, including pro-inflammatory cytokines, growth factors, and ECM components, thereby positioning them as modulators rather than passive executors of tissue remodeling. Moreover, inflammatory skin disorders are characterized by dysregulated protease networks, suggesting that meprins may contribute to the persistence of psoriatic inflammation (15, 16, 34, 81).

The ability of meprin proteins to activate IL-1 family cytokines may be a key argument for targeting them therapeutically, as they promote Th17 and Th1 differentiation, which contribute to psoriasis. In psoriasis, altered ECM composition contributes to leukocyte infiltration and aberrant keratinocyte behavior, implicating proteolytic imbalance in disease maintenance. By modifying the ECM structure and generating bioactive matrix fragments, meprins may indirectly shape immune cell positioning and activation in psoriatic lesions. Meprins might also contribute to psoriasis pathogenesis through their effects on immunological signaling and epithelial integrity, especially meprin β, which, when elevated, impairs syndecan-1 shedding (80).

From a therapeutic perspective, protease inhibition is challenging due to the lack of specificity and the toxicity associated with broad-spectrum metalloprotease inhibitors. However, meprin proteases possess unique active-site architectures and substrate preferences that distinguish them from MMPs and ADAM proteases, enabling selective targeting strategies (23, 31, 90). Structure-guided approaches have yielded small-molecule inhibitors with nanomolar potency and isoform selectivity for meprin α, demonstrating the feasibility of precise pharmacological modulation. Recent advances in the structural biology of meprin have further strengthened the therapeutic rationale for developing selective inhibitors. Unlike earlier broad-spectrum metalloprotease inhibitors that were limited by poor specificity and off-target toxicity, structure-guided approaches have enabled the design of small-molecule compounds that selectively target the unique active-site architecture of meprin isoforms. Such inhibitors provide an opportunity to suppress pathological cytokine maturation, ECM remodeling, and epidermal barrier disruption while minimizing interference with related metalloprotease families. Future studies could focus on optimizing isoform-selective compounds, particularly those targeting meprin α, which appears more directly linked to pathological epidermal remodeling and dermokine degradation in psoriasis. The development of topical formulations may further enhance safety by restricting inhibitor exposure to diseased skin and reducing systemic effects.

Selective inhibition of meprins offers conceptual advantages over blocking downstream cytokines. Although IL-17 and IL-23 inhibitors achieve high clinical efficacy, a subset of patients exhibits incomplete or transient responses, suggesting that tissue-based amplification loops remain active. Targeting meprin-mediated cytokine maturation and ECM remodeling could attenuate these upstream reinforcement mechanisms, potentially enhancing the depth and durability of the therapeutic response. Such an approach may also reduce dependence on systemic immunosuppression by modulating local inflammatory circuits within the skin (9, 10, 62, 91).

The function of endogenous inhibitors such as fetuin A and cystatin C suggests that partial, rather than total, meprin inhibition may be sufficient to restore proteolytic equilibrium (72). Therapeutic strategies that mimic or enhance endogenous inhibitory mechanisms can achieve disease modulation while preserving essential protease functions in tissue repair and host defense. An additional consideration for therapeutic development is the dual physiological and pathological role of meprins in cutaneous tissues. Although elevated meprin activity may contribute to inflammation and epidermal remodeling in psoriasis, complete inhibition could interfere with normal tissue homeostasis. Meprins participate in extracellular matrix processing, collagen maturation, and epithelial repair, and genetic loss-of-function models demonstrate impaired wound healing and altered matrix architecture in the absence of physiological meprin activity (15, 21, 33). Consequently, prolonged or non-selective inhibition may delay tissue repair or compromise barrier restoration following injury. Furthermore, the distinct biological functions of meprin α and meprin β suggest that isoform specificity will be critical for therapeutic success. Secreted meprin α appears more closely associated with epidermal remodeling and DMKN degradation, whereas membrane-bound meprin β contributes to adhesion molecule shedding and physiological epithelial regulation (18, 24, 34, 56). Selective targeting of disease-associated meprin α activity may therefore provide a wider therapeutic window than simultaneous inhibition of both isoforms. Since meprins are expressed in multiple organs, including the intestine and kidney, systemic administration may also increase the risk of off-target effects arising from disruption of physiological proteolytic processes outside the skin (14). These considerations support the development of localized topical delivery strategies and partial activity modulation approaches that preserve essential homeostatic functions while suppressing pathological proteolysis. Nevertheless, the therapeutic consequences of long-term meprin inhibition remain largely unexplored, as meprin-targeted inhibitors have not yet been evaluated in psoriasis models. Future studies are therefore required to define the therapeutic window, assess potential effects on wound healing and barrier restoration, and determine whether selective inhibition of disease-associated meprin activity can be achieved without compromising physiological tissue homeostasis.

The relatively restricted expression of meprins may limit off-target effects compared to broadly expressed metalloproteases. Despite these advantages, several challenges remain to be addressed. Protease networks exhibit redundancy, and inhibition of a single protease may induce compensatory upregulation of related enzymes (37, 72, 89). Furthermore, direct evidence linking meprin inhibition to clinical improvement in psoriasis is currently lacking, necessitating careful preclinical validation using skin-specific and immune-competent models. Activity-based proteomic profiling of psoriatic lesions is essential for defining patient subsets most likely to benefit from meprin-targeted interventions. Therefore, meprins are mechanistically plausible but underexplored therapeutic targets for psoriasis. Their ability to regulate cytokine activation, ECM remodeling, and epidermal integrity places them at the intersection of immune- and tissue-intrinsic disease mechanisms. Therapeutic use of meprins may complement existing immune-directed treatments by disrupting extracellular amplification loops that sustain chronic inflammation in psoriasiform skin (**Table 3****).**

**Table 3:** Therapeutic and regulatory strategies targeting meprins.

Approach	Representative strategy/ molecule	Mechanism of action	Therapeutic implication
Small molecule inhibitors	Selective meprin α/β inhibitors (e.g.: actinonin derivatives)	Bind to active site zinc motif; block cytokine activation	Reduce IL-1β and IL-18 maturation, dampen inflammation
Endogenous regulators	Fetuin A and Cystatin C	Inhibit proteolytic activity of meprins in extracellular space	Restores homeostatic protease balance
Epigenetic modulation	DNA methylation, histone acetylation, microRNAs (miR-155, miR-21)	Downregulate MEP1A/MEP1B gene expression	Attenuate chronic proteolytic activation in inflamed skin
Post translational regulation	Control of KLK mediated zymogen activation	Prevents premature meprin activation	Maintains epidermal protease homeostasis
Combination therapy potential	Meprin inhibitors + anti IL-17 biologics	Dual blockade of extracellular and immune activation	May enhance treatment durability and reduce relapse
Topical delivery systems	Nanocarriers, hydrogel-based formulations	Localize inhibitor to inflamed skin; minimize systemic exposure	Precision control of extracellular proteolysis

## 6 Future perspectives

Despite substantial advances in understanding meprin metalloproteases as regulators of extracellular proteolysis, many aspects of their immunological relevance remain unresolved. Existing models of chronic inflammation, including psoriasis, have largely focused on intracellular signaling while underappreciating the contribution of tissue-level enzymatic control. Furthermore, a comprehensive view of meprins requires integration across spatial, molecular, and functional scales, from their cell-specific activity to their roles in broader proteolytic and immune networks (15). The following priorities outline key conceptual and experimental directions that can bridge these gaps and translate meprin biology into therapeutic insights.

### 6.1 Integrating meprins into immune-centric disease frameworks

Although meprins are recognized as regulators of extracellular proteolysis in inflammatory settings, their integration into immune-centric disease models remains incomplete. Current immunological frameworks for chronic inflammatory disorders, including psoriasis, predominantly emphasize cytokine signaling and immune cell differentiation. However, extracellular proteolytic control is now recognized as a parallel axis shaping immune behavior (5, 15). Future research must reposition meprins within system-level models that account for the tissue context, protease network interactions, and substrate dynamics.

### 6.2 Defining cellular and compartment-specific functions

A key priority is to delineate the cell-type- and compartment-specific roles of meprin α and β in inflamed tissues. Transcriptomic data indicate substantial divergence in protease expression between epithelial, stromal, and immune compartments; however, bulk profiling obscures this heterogeneity. The application of spatial transcriptomics and single-cell proteomics to diseased skin will clarify the location and timing of meprin’s immunomodulatory effects relative to the adaptive immune niche (37, 89). Such resolution is critical because extracellular proteolysis may yield opposing outcomes depending on the spatial context and substrate accessibility.

### 6.3 Mapping the *in-vivo* substrate landscape

Another frontier involves defining the *in vivo* substrate spectrum of meprin under inflammatory conditions. While several cytokines and ECM components have been validated as meprin substrates *in vitro*, their activity *in situ* is shaped by competing substrates, inhibitors, and spatial restrictions (35). Activity-based protein profiling and quantitative degradomics are essential for identifying direct cleavage events in inflamed skin and may reveal non-canonical substrates linking proteolysis to immune cell recruitment.

### 6.4 Structural insights and rational inhibitor design

Recent advances in structural and chemical biology have opened new avenues for selective modulation of meprin. Structural elucidation of meprin α oligomerization and active-site architecture highlights features that distinguish meprin from other metzincins (27, 31). These insights enable the rational design of isoform-selective inhibitors that avoid the toxicity historically associated with broad-spectrum metalloprotease blockade. The integration of high-resolution crystallography with computational ligand screening can accelerate the discovery of compounds with favorable selectivity profiles. However, inhibitor development must proceed alongside biological validation in immune-competent models. Protease inhibition can trigger compensatory remodeling within protease networks, potentially undermining therapeutic efficacy. Therefore, system-level analyses of protease-protease interactions will be critical for anticipating adaptive responses and designing combination strategies that modulate entire proteolytic circuits rather than single enzymatic nodes.

### 6.5 Harnessing endogenous regulatory mechanisms

In addition to small-molecule inhibitors, endogenous regulatory systems provide alternative therapeutic paradigms. Macromolecular inhibitors and post-translational modifications that fine-tune proteolysis physiologically restrain meprin activity. Enhancing these natural controls could permit partial, context-specific suppression of pathological proteolysis while preserving essential tissue remodeling (14, 72). Such strategies reflect modern immunological approaches favoring modulation over ablation of immune-relevant pathways.

### 6.6 Biomarkers and clinical translation

Translating meprin biology into clinical settings will require the development of biomarkers for patient stratification and pharmacodynamic monitoring. Protease-derived cleavage fragments and activity signatures serve as direct indicators of tissue-level responses (72, 80). Longitudinal skin sampling and non-invasive biomarker assays may enable the precise deployment of protease-targeted interventions. Localized drug delivery further enhances the translational feasibility. Nanostructured topical systems or controlled-release formulations can confine inhibitor exposure to diseased skin, thereby minimizing systemic effects. Such strategies are particularly suited for extracellular enzymes, such as meprins, whose activity is spatially restricted.

Beyond their therapeutic relevance, meprins may also serve as biomarkers of disease activity and treatment response (42). Altered expression of meprin α, changes in proteolytic activity signatures, and the generation of characteristic cleavage products derived from cytokines, ECM proteins, or epidermal substrates such as dermokine may provide measurable indicators of ongoing tissue remodeling and inflammatory activity. Integration of activity-based proteomics, degradomics, and spatial profiling approaches could facilitate patient stratification and identify individuals most likely to benefit from meprin-targeted interventions. Such biomarkers may also enable longitudinal monitoring of therapeutic efficacy and help distinguish responders from non-responders in future clinical studies.

### 6.7 Integrative models and systems immunology

Advancing meprin research requires an expanded immunological framework that integrates tissue biology as an active determinant of immune regulation. Extracellular proteolysis shapes cytokine gradients, ECM stiffness, and cell-cell communication, all of which influence adaptive immune responses. Recognizing meprins within this extended landscape highlights proteolytic regulation as a unifying principle across chronic inflammatory diseases (34, 42, 81). Coordinated efforts spanning structural biology, systems immunology, proteomics, and translational science are essential to address the current gaps in spatial resolution, substrate mapping, and network integration. Embedding meprins within tissue immune crosstalk frameworks may ultimately yield new strategies to modulate chronic inflammation beyond conventional cytokine-centric paradigms.

## 7 Conclusion

Meprins are emerging as vital yet underestimated regulators at the junction of extracellular proteolysis, tissue architecture, and immunological signaling. Previously thought to be localized epithelial enzymes, they have now been identified as context-dependent mediators that influence cytokine activation, matrix remodeling, and intercellular communication within inflamed tissues. They maintain inflammation without relying on classical intracellular pathways, offering a paradigm for understanding persistent immune activation in disorders such as psoriasis. They are influenced by spatial confinement, endogenous inhibitors, and post-translational regulation, resulting in a proteolytic network that links structural remodeling to immunological and metabolic adaptability. The therapeutic issue is not silencing meprin but rather restoring balance through targeted, spatially constrained regulation that protects physiological remodeling while attenuating inflammation. Advances in structural biology and chemical design have made the development of isoform-selective small-molecule meprin inhibitors increasingly feasible, offering a potential strategy to target upstream extracellular amplification mechanisms in psoriasis. In parallel, meprin-associated proteolytic signatures and substrate-derived cleavage products may evolve into clinically useful biomarkers for disease stratification, pharmacodynamic monitoring, and assessment of therapeutic response. Together, these advances strengthen the translational potential of meprins as both therapeutic targets and biomarker candidates in inflammatory skin disease. Integrating meprin biology into systems-level models of immunity has the potential to reshape our understanding of extracellular enzymatic activities as dynamic, regulatory factors involved in immunological homeostasis and chronic illness. This would help open new avenues in the biological arena.

## Glossary

ADAM: A Disintegrin and Metalloproteinase
AP-1: Activator Protein-1
APCs: Antigen Presenting Cells
APP: Amyloid Precursor Protein
BSB: Blood–Skin Barrier
DAMPs: Damage Associated Molecular Patterns
DMKN: Dermokine
DNA: Deoxyribonucleic Acid
ECM: Extracellular Matrix
EGF: Epidermal Growth Factor
IL: Interleukin
KLK: Kallikrein Related Peptidase
LPS: Lipopolysaccharide
MAM: Meprin A5 Protein Tyrosine Phosphatase µ Domain
MMPs: Matrix Metalloproteinases
MT-MMP: Type Matrix Metalloproteinase
NFκB: Nuclear Factor Kappa Light Chain Enhancer of Activated B Cells
PRRs: Pattern Recognition Receptors
RNA: Ribonucleic Acid
STAT-3: Signal Transducer and Activator of Transcription 3, TGF, Transforming Growth Factor
TIMPs: Tissue Inhibitors of Metalloproteinases
TNF: Tumor Necrosis Factor
TRAF: Tumor Necrosis Factor Receptor Associated Factor
UUO: Unilateral Ureteral Obstruction
VEGF: Vascular Endothelial Growth Factor
